# Bowenoid Actinic Keratosis in a Patient Receiving Abatacept for Rheumatoid Arthritis: A Case Report

**DOI:** 10.7759/cureus.95856

**Published:** 2025-10-31

**Authors:** Maya Kaga, Hirofumi Amano, Takayuki Kon, Kanako Ogura

**Affiliations:** 1 Department of Dermatology, Juntendo University Nerima Hospital, Tokyo, JPN; 2 Department of Rheumatology and Internal Medicine, Juntendo University Nerima Hospital, Tokyo, JPN; 3 Department of Pathology and Laboratory Medicine, Juntendo University Nerima Hospital, Tokyo, JPN

**Keywords:** abatacept, actinic keratosis, bowenoid actinic keratosis, imiquimod, rheumatoid arthritis, toll-like receptors

## Abstract

We experienced a case of Bowenoid actinic keratosis on the left wrist joint in a patient receiving abatacept for rheumatoid arthritis. Simple treatment with imiquimod, initiated with the continuous use of abatacept, did not improve the lesion. Topical imiquimod successfully cured the lesion only after abatacept discontinuation in the patient. Our case suggests that abatacept has depleted the production of tumor necrosis factor-α by macrophages upon contact with imiquimod, which is a toll-like receptor 7 ligand.

## Introduction

Toll-like receptors (TLRs), which belong to the protein recognition receptors, are related to innate immunity reactions [1]. In patients with rheumatoid arthritis (RA), it is described, abnormal presence of TLR-ligands, altered responses to infectious agents, and increased production of pro-inflammatory cytokines exist in the peripheral blood and synovial fluid, possibly through their genetic and epigenetic factors [1]. Joint damages, bone erosions, and cartilage destruction were suggested to be the result of TLR2/3/4/6/7/9-dependent second hit mechanism at the joint [1]. These studies support the treatment strategy for RA with modulators for TLR-ligands such as disease-modifying antirheumatic drugs (DMARDs), including abatacept.

Among the TLR family, TLR7 exists in endosomes of monocytes, macrophages, dendritic cells, and mast cells, together with TLR8 and 9. In particular, Chamberlain et al. suggested TLR7 as a predictor of the severity of RA, as increased expression of TLR7 was observed in the synovial tissue of patients with RA. [2] However, the direct link between abatacept and TLR7 signaling in the skin tissue remains to be fully elucidated.

Actinic keratoses (AK), usually predominant in a sun-exposed distribution, are accepted as squamous cell carcinoma (SCC) in situ. RA is an independent risk factor for nonmelanoma skin cancers (NMSCs), including AK, basal cell carcinoma, SCCs, and Bowen’s disease [3]. Additionally, the identification of treatments that increase the risk of NMSC in patients with RA remains controversial. While some studies suggested that NMSC risk may be higher with abatacept use compared to DMARDs [4], Scott et al. reported in a retrospective cohort study that abatacept did not increase the incidence of NMSC in patients with RA [5]. We present here a case of AK in a patient with RA identified after abatacept initiation.

## Case presentation

**Figure 1:**
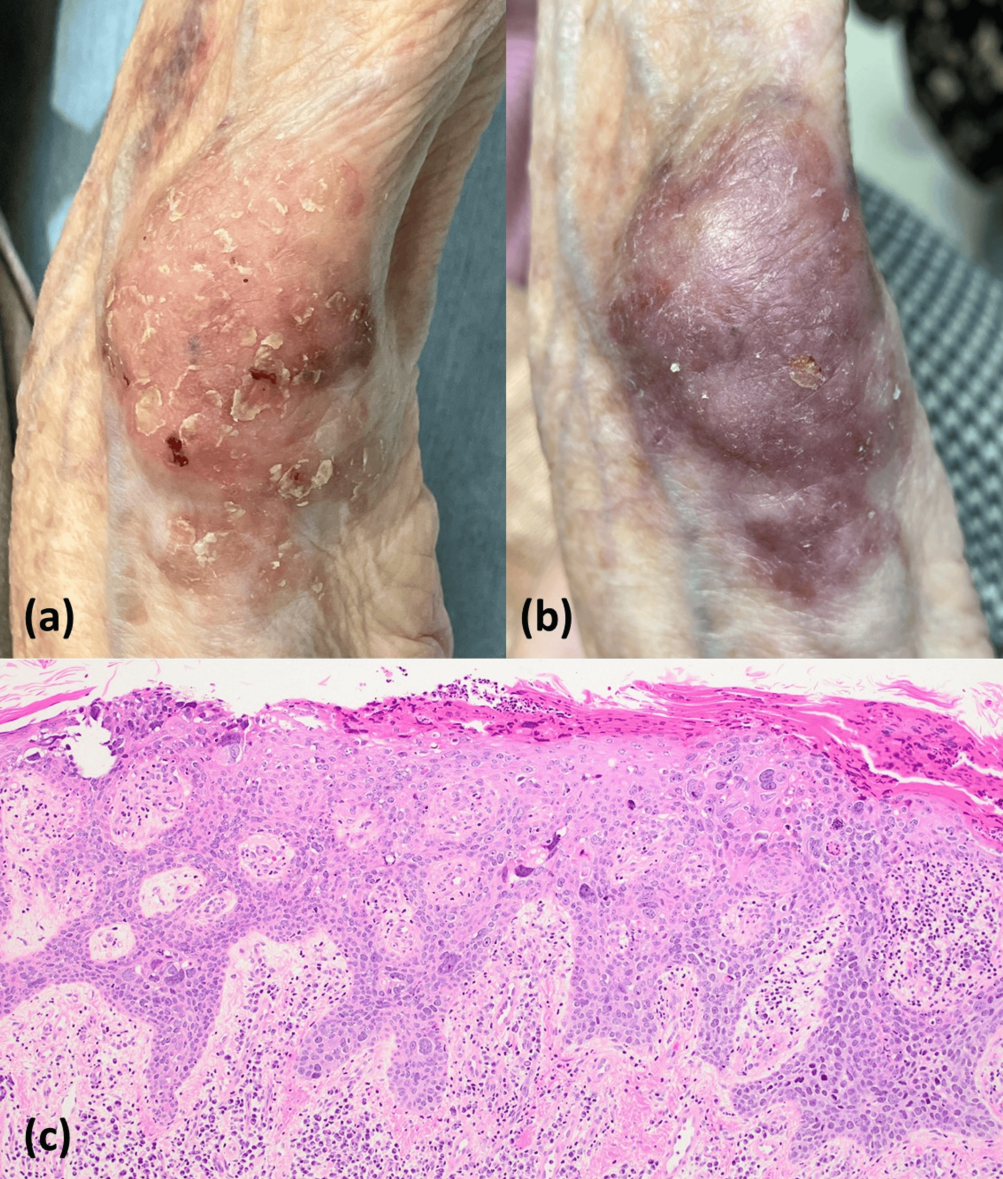
Clinical and histological features of the skin lesion. (a) Erythematous and keratotic macule on the ulnar dorsal side of the left wrist joint. (b) The macule became dark or purpuric one month after imiquimod was initiated, while abatacept therapy was continued. (c) Histology of the skin biopsy shows atypical keratinocytes in the epidermis with clumping cells, accompanied by dermal solar elastosis and inflammatory cells, as observed with hematoxylin and eosin staining (×100).

A 90-year-old Japanese woman presented with a scaly macule on her left wrist joint one to two years before presentation. Her medical history included significant RA since the age of 85 years. She had previously been treated with salazosulfapyridine (DMARD), iguratimod (DMARD), and prednisolone, and over the past 15 months, she had received oral prednisolone 2 mg/day along with weekly subcutaneous injections of abatacept 125 mg. She had noticed skin pigmentation since the age of 75 years, which she recognized was the result of daily sun exposure. An erythematous, hyperkeratotic, partially crusted macule measuring 4.5 × 3.5 cm was observed (Figure 1a).

Skin biopsy revealed atypical keratinocytes in all layers of the epidermis. Clumping cells, dermal solar elastosis, and inflammatory cells were observed (Figure 1c). The patient was diagnosed with Bowenoid actinic keratosis. Simple treatment with imiquimod 12.5 mg, used topically for 12 hours, three times per week, was initiated with the continuous use of abatacept. Four weeks after the initiation of the imiquimod treatment, the lesion became darkened, purpuric, and slightly edematous (Figure 1b). Therefore, our patient was treated with topical imiquimod for an additional two months with abatacept discontinuation, together with increased doses of salazosulfapyridine for RA. When the lesion disappeared two months later, she was transferred to another hospital with continuous termination of abatacept. Her family says there is no sign of tumor relapse after the treatment.

## Discussion

Imiquimod, a TLR7 ligand, is the standard treatment for AK. Imiquimod is indicated to reverse the abnormal expression of some of the gene profiles localized in AK, in the cluster analysis of aberrant gene expression between pretreatment and post-treatment groups [6]. They speculated that these gene expression patterns would lead to attenuated regulation of cell proliferation in AK [6]. On the other hand, abatacept is a soluble protein, CTLA4 with an Fc tail, which binds to CD80 and CD86 on the antigen-presenting cells. These connecting blocks T-cell instruction by antigen-presenting cells [7]. Abatacept depletes the production of tumor necrosis factor-α by macrophages; consequently, it induces cytokine-activated T cells and TLR ligands in the peripheral blood cells of healthy humans [7].

Depending on these characterized mechanisms, we speculated that abatacept possibly and partially tolerates TLR7 ligand-induced activation of T cells in the skin tissue of RA patients. A unique color change in this case (Fig.1b) suggested that an imiquimod-induced cellular inflammatory reaction and cytokine production to dysplastic keratinocytes in the lesional skin were insufficient or modified in this case because of the background of abatacept.

The skin lesions disappeared after three months of imiquimod treatment - one month with abatacept and two months without abatacept. We suspected an AK lesion in this case, which may have resolved with imiquimod treatment or regressed spontaneously after discontinuation of abatacept, considering that spontaneous regression of AK has been rarely reported in Asia [8]. Although there is a lack of evidence in the literature showing that abatacept induces NMSC lesions, we decided not to use abatacept again in this case to prevent multiple AKs.

## Conclusions

There are potential limitations of clinical data in our case. We could not persuade a long-time follower, because the patient was transferred to another hospital. We cannot conclude that abatacept therapy induced AK or limited the treatment effect for AK, because a second biopsy was not necessarily needed for her treatment. However, our case showed the possibility that abatacept may have attenuated the effects of imiquimod topically used for AK in an old patient with RA. We believe our case brings some insights; the effect of topical imiquimod was influenced by the status of immune responses of the patient. We can recommend immediate withdrawal of abatacept during imiquimod administration as long as the status of RA is tolerable with alternative treatment options.
